# Supplementary material for: Microscaled Cell Surface Proteomics for Cryo-preserved Cells and Tissue Samples
Source: bioRxiv. 2025 Jul 20:2025.07.18.664488. Preprint. [Version 2] doi: 10.1101/2025.07.18.664488 (PMC12338646; doi:10.1101/2025.07.18.664488)
Supplement: 1 [file NIHPP2025.07.18.664488V2-supplement-1.pdf]

bioRxiv preprint doi: https://doi.org/10.1101/2025.07.18.664488; this version posted July 20, 2025. The copyright holder for this preprint (which was not certified by peer review) is the author/funder, who has granted bioRxiv a license to display the preprint in perpetuity. It is made available under aCC-BY 4.0 International license.

Figure S1

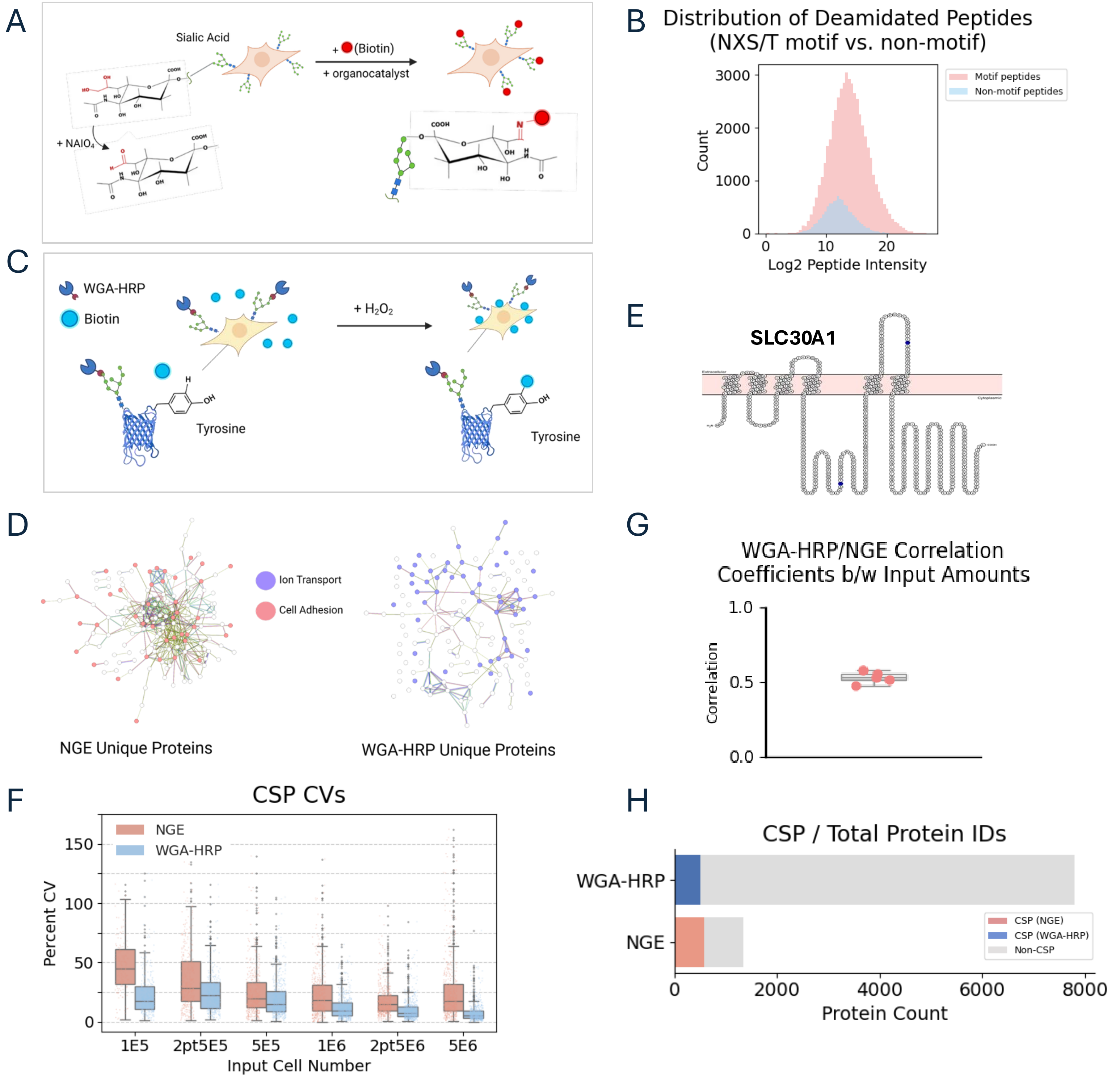

Figure S1. Comparative strategies for cell surface protein labeling and additional HEK titration metrics **A.** Cell surface glycan labeling by $NaIO_4$ oxidation and amine-based biotinylation. Sialic acid residues are selectively oxidized to aldehydes and reacted with an amine-containing biotin reagent in the presence of an organocatalyst, 5-methoxyanthranilic acid (5MA). **B.** Log_2-transformed peptide intensity distributions for motif-containing and non-motif peptides from N-glyco–enriched samples (across all HEK293T inputs, includes overlapping peptides between inputs) were classified based on confident deamidation site localization (localization probability ≥ 0.75) and N-X-S/T motif presence, with motif-containing proteins retained for downstream CSP analysis. **C.** Cell surface protein labeling by WGA-HRP and biotin-tyramide. WGA-HRP binds to terminal GlcNAc and sialic acids, enabling HRP-catalyzed deposition of biotin-tyramide onto proximal tyrosine residues. **D.** STRING network analysis of proteins uniquely identified by each method, annotated for functions in cell adhesion and ion transport with BH FDR < 0.01. **E.** Representative topology of the ion transport protein SLC30A1, uniquely identified by WGA-HRP. The diagram was generated using the Protter web-based application, with blue dots indicating N-X-S/T motifs. **F.** Coefficient of variation (CV) percentages for fully quantified proteins across replicates at each input amount, assessing quantitative reproducibility (N = 3). **G.** Pearson correlation coefficients between glycan oxidation– and WGA-HRP–based surfaceome data across input amounts (1×10^5 to 5×10^6 cells). **H.** Cell surface protein identifications compared to total number of proteins cumulatively across all HEK titration input amounts for both protocols.

bioRxiv preprint doi: https://doi.org/10.1101/2025.07.18.664488; this version posted July 20, 2025. The copyright holder for this preprint (which was not certified by peer review) is the author/funder, who has granted bioRxiv a license to display the preprint in perpetuity. It is made available under aCC-BY 4.0 International license.

Figure S2

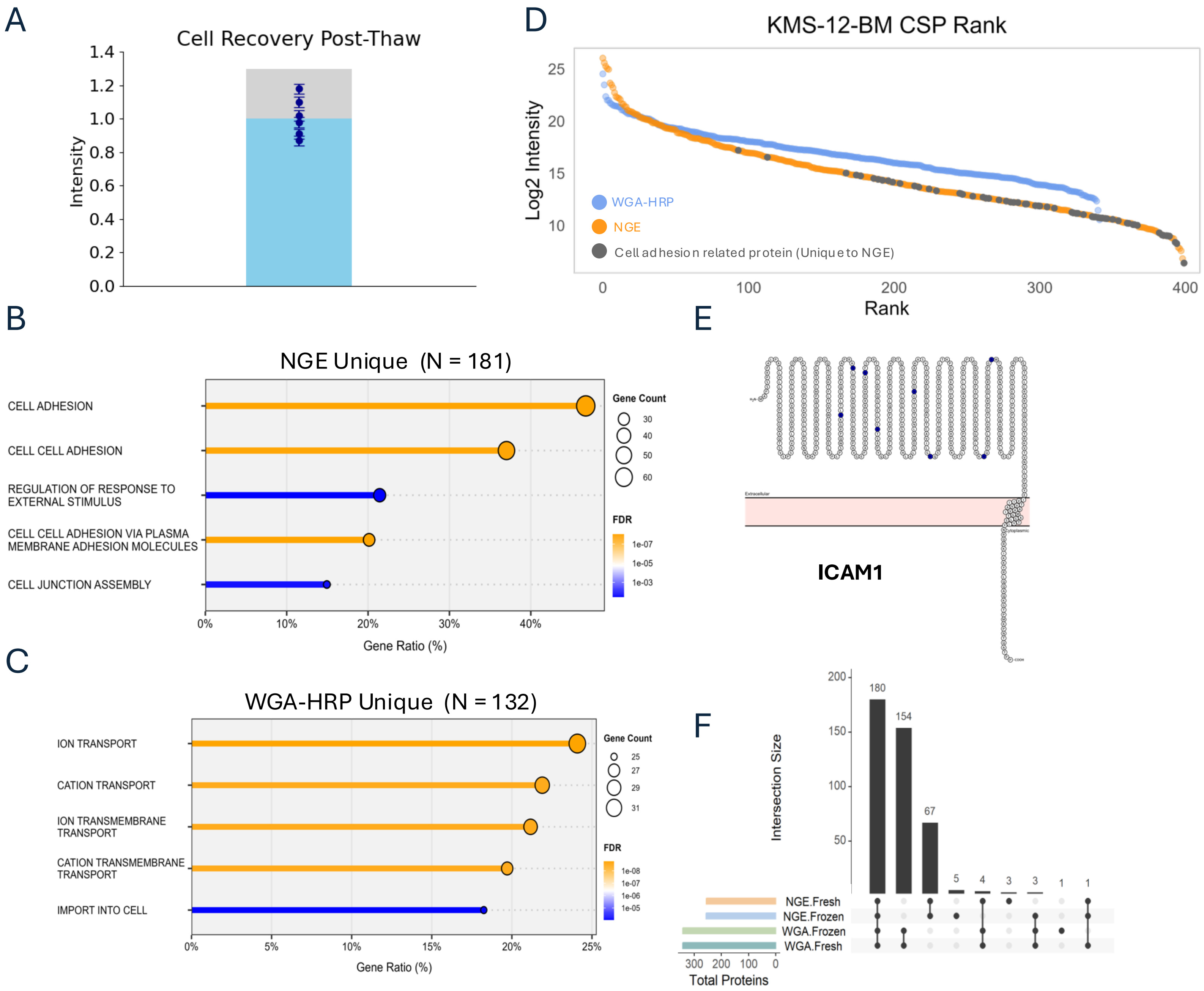

Figure S2. Method-specific surface proteome features and agreement metrics for KMS-12-BM suspension cell line A. Bar plot of post-thaw cell recovery, with pre-freeze cell count (1.3 × 10^6) shown as a light gray background bar. Average cell recovery was 1.1 × 10^6 (N = 6). **B–C.** GO Biological Process overrepresentation analysis for proteins uniquely identified by each method. Enriched terms for NGE-unique proteins are dominated by cell adhesion processes; ion transport processes are enriched for WGA-HRP–unique proteins. **D.** Rank plots showing the dynamic range of surface protein intensities for each enrichment method in KMS-12-BM cells. **E.** Representative topological map of a cell adhesion protein identified by N-glyco enrichment (ICAM1), highlighting a large extracellular domain with multiple predicted N-glycosylation motifs. The diagram was generated using the Protter web-based application, with blue dots indicating N-X-S/T motifs. **F.** Upset plot showing the number of intersecting proteins among fresh and frozen samples labeled with either NGE or WGA-HRP. Horizontal bars represent total number of proteins identified in each dataset.

bioRxiv preprint doi: https://doi.org/10.1101/2025.07.18.664488; this version posted July 20, 2025. The copyright holder for this preprint (which was not certified by peer review) is the author/funder, who has granted bioRxiv a license to display the preprint in perpetuity. It is made available under aCC-BY 4.0 International license.

Figure S3

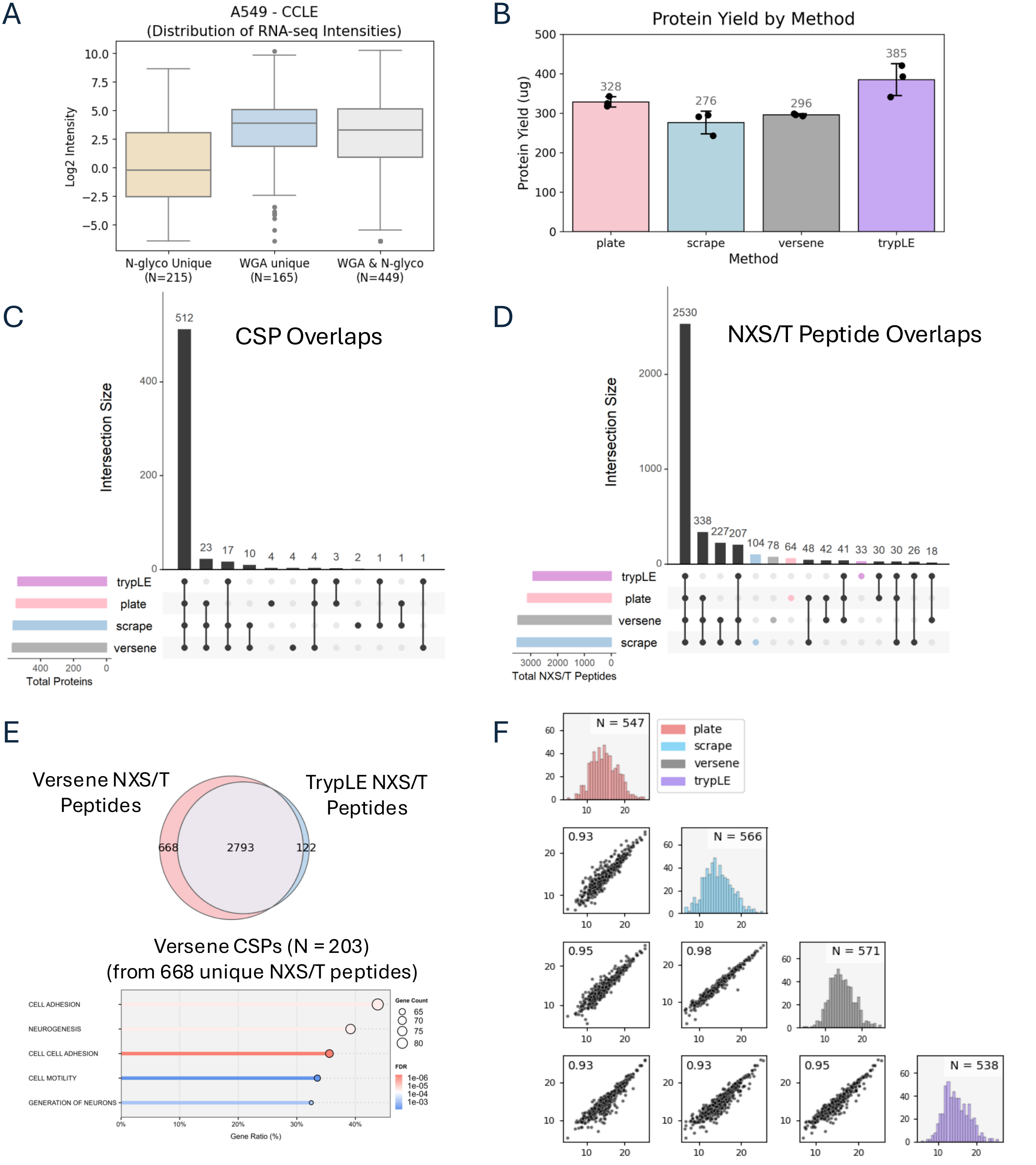

Figure S3. Method-specific surface proteome features and agreement metrics for A549 adherent cell line
A. RNA expression levels (log_2 intensity) for surface proteins uniquely identified by N-glyco enrichment (N = 215), WGA enrichment (N = 165), or detected by both methods (N = 449) in A549 cells, using CCLE RNA-seq data. **B.** Total protein yield (µg) across four dissociation methods (plate, scrape, versene, trypsin-based TrypLE) from equal cell inputs. Each dot represents an independent replicate. **C.** Overlap of cell surface proteins (CSPs) identified across the four dissociation methods. The largest shared set (N = 516) is common to all methods, with smaller method-specific subsets. **D.** Overlap of identified NXS/T-containing peptides across the four dissociation methods. **E.** Gene Ontology Biological Process (GO:BP) over-representation analysis of CSPs detected from the versene condition (N = 210) within the 696 unique NXS/T peptides. Top enriched terms include neurogenesis, cell adhesion, and motility.
F. Scatter plots and histograms showing consistent surface protein quantification across A549 6well plate conditions (plate-based labeling and various cell detachments) for the NGE method.

bioRxiv preprint doi: https://doi.org/10.1101/2025.07.18.664488; this version posted July 20, 2025. The copyright holder for this preprint (which was not certified by peer review) is the author/funder, who has granted bioRxiv a license to display the preprint in perpetuity. It is made available under aCC-BY 4.0 International license.

Figure S4

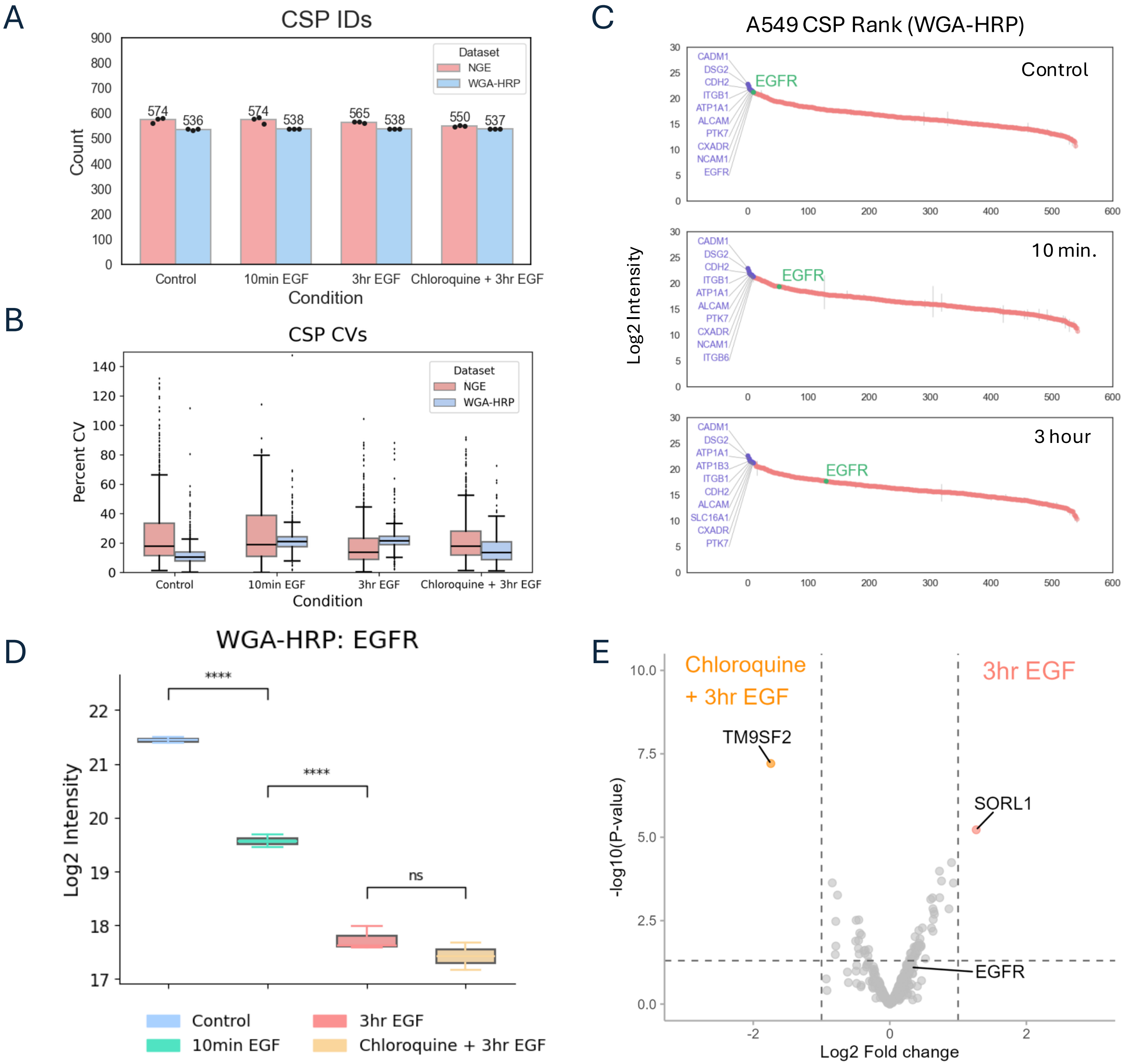

Figure S4. Method-specific surfaceome performance and EGFR detection across receptor trafficking conditions
A. Surface protein identifications across the four treatment conditions from NGE and WGA-HRP–based surfaceome profiling (N = 3). **B.** Coefficients of variation (CVs) across the four treatment conditions from NGE and WGA-HRP–based surfaceome profiling (N = 3). **C.** Rank plot of WGA-HRP–based surfaceome data from control, 10 min EGF, and 3 hr EGF conditions, highlighting EGFR and high-abundance stable surface proteins. **D.** EGFR abundance measured by WGA-HRP across the four conditions, showing consistency with NGE-based trends (N=3). Significance determined by unpaired two-tailed t-tests: $P > 0.05$ (NS), $P < 0.0001$ (****). **E.** Volcano plot of WGA-HRP surfaceome data comparing 3-hour EGF vs. chloroquine + 3-hour EGF, showing downregulation of EGFR recycling proteins in chloroquine-pretreated samples, with less pronounced EGFR suppression—potentially due to intracellular EGFR contributing to background signal ($\log_2$-fold change > |1|; p < 0.05 by t-test).

bioRxiv preprint doi: https://doi.org/10.1101/2025.07.18.664488; this version posted July 20, 2025. The copyright holder for this preprint (which was not certified by peer review) is the author/funder, who has granted bioRxiv a license to display the preprint in perpetuity. It is made available under aCC-BY 4.0 International license.

Figure S5

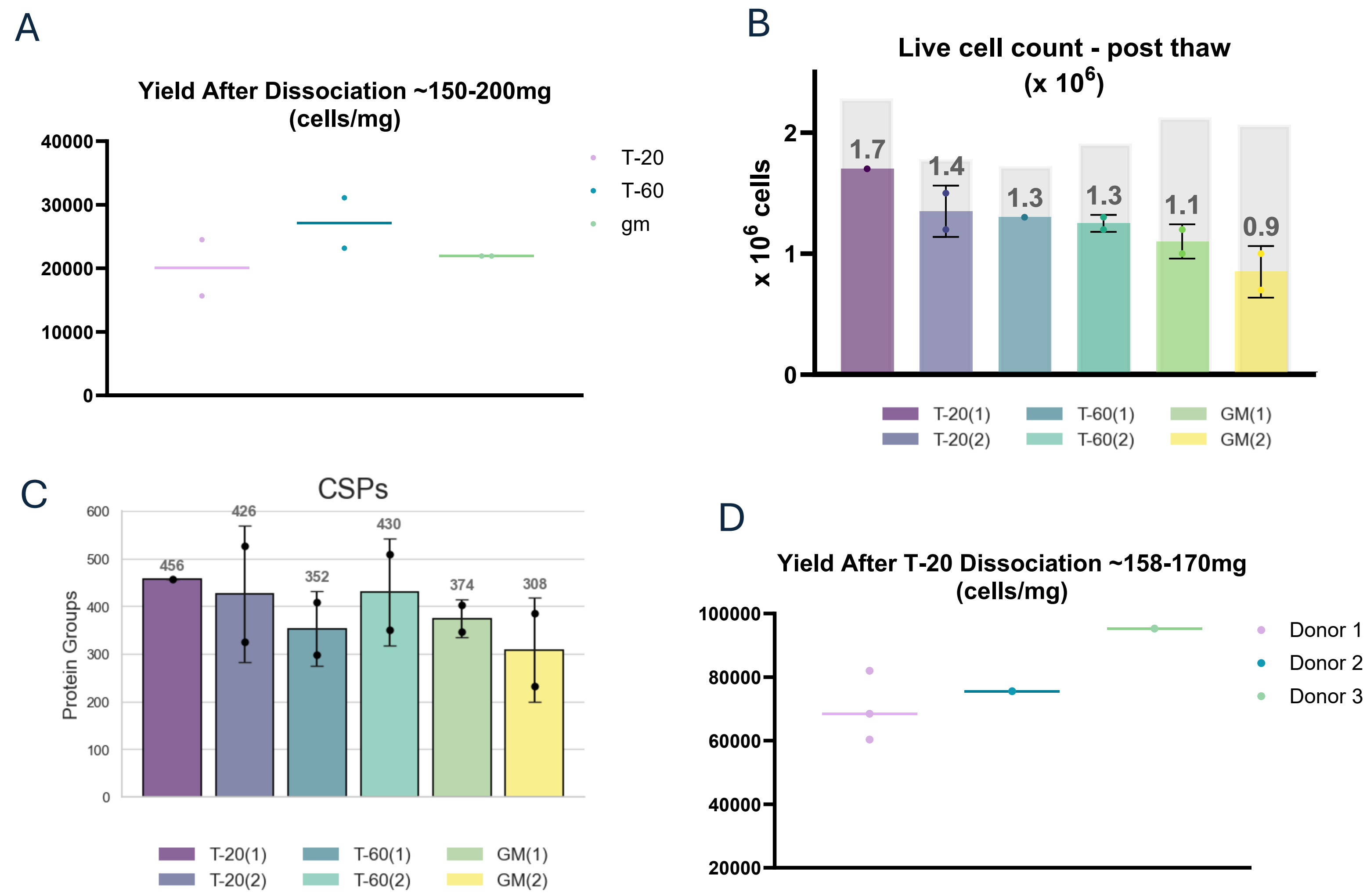

Figure S5. Evaluation of tissue dissociation strategies for cryopreserved surface proteomics
A. Workflow for dissociation and cryopreservation of fresh endometrial tissue using three protocols: T-20, T-60, and gentleMACS (GM). **B.** Post-thaw live cell counts following cryopreservation. Bars show the number of viable cells recovered after thawing, based on two biological replicates per condition (e.g., T-20(1), T-20(2)), each derived from independent tissue pieces. Error bars represent replicate cryopreserved vials from the same dissociation, processed and sorted with matched cell numbers. Light gray bars indicate live cell counts prior to cryopreservation. **C.** Number of confident cell surface proteins (CSPs) identified across all cryopreserved samples. Each bar represents a biological replicate; error bars show technical replicate variability following surface enrichment and MS analysis. **D.** Cell yields obtained during T-20 dissociation of three independent donor samples (158–170 mg tissue per sample), used in downstream titration experiments. Cell recovery ranged from ~60,000 to 90,000 cells per mg of tissue.
